# Bio-Inspired Covert Active Sonar Strategy

**DOI:** 10.3390/s18082436

**Published:** 2018-07-26

**Authors:** Jiajia Jiang, Xianquan Wang, Fajie Duan, Chunyue Li, Xiao Fu, Tingting Huang, Lingran Bu, Ling Ma, Zhongbo Sun

**Affiliations:** The State Key Lab of Precision Measuring Technology and Instruments, Tianjin University, Tianjin 300072, China; jiajiajiang@tju.edu.cn (J.J.); fjduan@tju.edu.cn (F.D.); 2016202086@tju.edu.cn (C.L.); fuxiao215@tju.edu.cn (X.F.); huangtingting@tju.edu.cn (T.H.); lingranbu@tju.edu.cn (L.B.); tinama_17116@tju.edu.cn (L.M.); zbsun@tju.edu.cn (Z.S.)

**Keywords:** active sonar, sonar waveform design, bio-inspired sonar, covert sonar

## Abstract

The covertness of the active sonar is a very important issue and the sonar signal waveform design problem was studied to improve covertness of the system. Many marine mammals produce call pulses for communication and echolocation, and existing interception systems normally classify these biological signals as ocean noise and filter them out. Based on this, a bio-inspired covert active sonar strategy was proposed. The true, rather than man-made sperm whale, call pulses were used to serve as sonar waveforms so as to ensure the camouflage ability of sonar waveforms. A range and velocity measurement combination (RVMC) was designed by using two true sperm whale call pulses which had excellent range resolution (RR) and large Doppler tolerance (DT). The range and velocity estimation methods were developed based on the RVMC. In the sonar receiver, the correlation technology was used to confirm the start and end time of sonar signals and their echoes, and then based on the developed range and velocity estimation method, the range and velocity of the underwater target were obtained. Then, the RVMC was embedded into the true sperm whale call-train to improve the camouflage ability of the sonar signal-train. Finally, experiment results were provided to verify the performance of the proposed method.

## 1. Introduction

By sending out signals for target detection, an active sonar system [1,2,3,4,5] unavoidably risks being detected and identified by the others too. In the last few decades, many methods have been proposed to improve the covert capability of active sonar systems through signal waveform design [6,7,8,9,10,11,12,13,14,15,16] from the perspectives of low probability of interception (LPI) or low probability of detection (LPD).

Some methods try to constantly change the parameters of the transmitted signals to increase the identification difficulty, such as period-hopping [6], frequency-hopping [7,8], time-hopping [9], and so on. Although they can improve the covertness of signals, there is still much further work to be done, since those changed-parameters signals have some distinct features. For example, for frequency-hopping signals, each pulse could be continuous-wave (CW) or linear frequency modulated (LFM); however, the CW pulses have the feature of being of rectangle in the time domain and single frequency in the frequency domain, while LFM pulses are of both rectangle in the time domain and linearly changed frequency in the frequency domain [10]. As a result, these signals can be identified and classified easily in practice [15,16].

Some other methods use low signal noise ratio (SNR) signals with LFM [10], FM-CW [11,12], or other stealth signals, such as wide spectrum low power pseudorandom [13,14] or chaotic codes [14] to increase the difficulty of being detected. Achieving a high degree of covertness, these signals can still be detected by some methods, such as envelope detection, energy detection, energy spectral density analysis methods, etc. [17,18]. In addition, in the underwater environment, the application of these signals is enslaved to the bandwidth constrained underwater acoustic channel and limited bandwidth underwater acoustic transmitter. Besides, a sonar system using the signals with low SNR requires a long-time energy accumulation process for target detection, which severely affects the detection efficiency.

Due to the similarity of considered problems, many ideas in radar signal waveform design [19,20,21] can also be employed here, and for the specific covert signal waveform design problem, those ideas in radar also fall into the above two categories [8,13].

Another direction for sonar waveform design is the nature-inspired approach. Given nature’s ability to address complex, large-scale problems with robust, adaptable, and efficient solutions resulting from many years of evolution, researchers look to natural systems for inspiration and methods to solve problems in human-created artificial environments. Based on the bio-sonar systems in nature, Rolf Muller et al. present a detailed review and discussion about bio-inspired engineering, and suggests that people learn and utilize some strengths of bio-inspired engineering to serve practical engineering applications [22]. Chris Capus et al. designed a novel and interesting bio-inspired wideband sonar signal waveform based on the double down-chirp structure of clicks of bottlenose dolphin and evaluated its performance [23]. Inspired by bats, Martin Hurtado develops an adaptive algorithm for joint design of the optimal waveform and trajectory of the moving radar [24]. This algorithm effectively increases the amount of collected information, reduces power consumption and computation load, and reduces tracking errors. Michele Vespe et al. introduced a range of strategies employed by bats and considered how these might be exploitable in future radar systems [25]. These bio-inspired ideas and in-depth analyses are very interesting and inspirational. However, to our best knowledge, the covertness issue has not been considered yet in this context. Eran Amichai et al. studied how bats detect and recognize the faint echoes generated by their own calls when many individuals emit bio-sonar calls simultaneously, and obtained an important and novel achievement: the bats’ response aimed to increase the signal-to-noise ratio and not to avoid spectral overlap [26]. In their study, they utilize the original bat call pulses or those man-made call pulses, which are generated by changing the distribution of spectral energy of the original bat call pulses, and artificial ICI to mimic the bat calls so as to test the bats’ response. Their mimicking method for biomimetic bat calls is inspirational.

**Figure 1:**
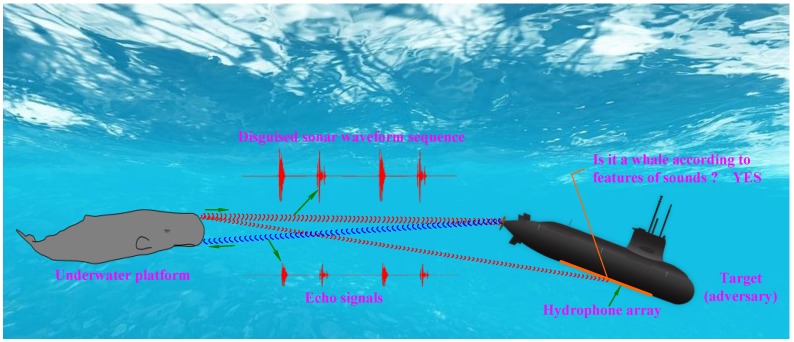
Bio-inspired covert active sonar strategy.

As can be seen in previous papers [27,28,29], these studies present interception systems that almost always classify biological signals as ocean noise and try to filter them out. Based on this, we propose a bio-inspired covert active sonar strategy. The true sperm whale call pulses with excellent RR and large DT are screened out to serve as sonar waveforms. A range and velocity measurement combination (RVMC) composed of two true sperm whale call pulses, which has excellent RR and large DT, was designed. Single call pulse in each RVMC was used to measure the range of the target, and based on each RVMC, a velocity measurement method was developed to measure the velocity of the target. In order to improve the camouflage ability of sonar waveform sequences, the RVMC are embedded into the true sperm whale call-train to hide the sonar waveforms. The proposed bio-inspired covert active sonar strategy is shown in Figure 1.

The main contributions of this paper can be summarized as follows:

(1) Different from conventional parameter-changing or low SNR sonar signal waveforms, and not to construct bionic sonar waveform by imitating the time domain waveform and time-frequency spectrum of the true sperm whale call pulses, the true sperm whale call pulses with excellent RR and large DT are used to serve as sonar waveforms, which can ensure that the sonar waveforms are not man-made but come from nature and thus have very good camouflage ability.
(2) A computationally efficient target range and speed measurement algorithm employing the characteristics of time resolution and Doppler tolerance of sonar waveforms was developed.
(3) Because the signal-train transmitted by the active sonar system is composed of the true sperm whale call-train, which is embed by sonar waveforms, the signal-train is very close to the true sperm whale call-train, and thus is difficult to classify into sonar signals rather than a marine mammals’ sound. So the signal-train transmitted by the active sonar system can obtain excellent camouflage ability.
(4) The proposed approach overcomes the trade-off between long-range detection and covertness. It can obtain covertness camouflage even if the SNR of the transmitted signals is very high. On the other hand, it can improve the covertness by reducing the SNR for a short range target detection task.

It is noted that the sperm whale is one of many kinds of whales and can be found anywhere in the open ocean [30,31,32,33,34]. Their distribution demonstrates that the sonar system using their call pulses to serve as the sonar waveforms can be used widely in any open sea area and thus has good generality.

## 2. Bio-Inspired Disguised Active Sonar Strategy Based on Sperm Whale Calls

### 2.1. The Characteristics and Laws of Sperm Whale Call-Train

In order to accurately imitate the sperm whale call-train for covert (disguised) sonar waveform sequence construction, firstly, we must look into the characteristics and laws of sperm whale call-trains.

**Figure 2:**
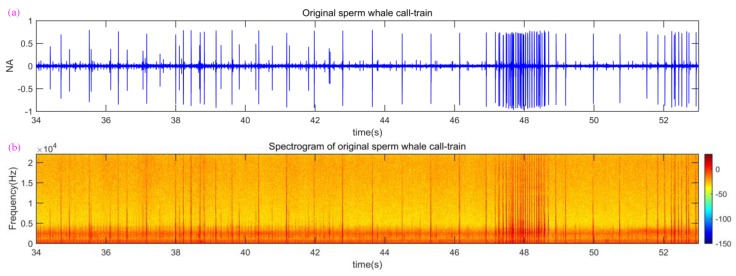
Waveform and spectrogram of original sperm whale call-train from the 34th to the 53rd s. (**a**) Original sperm whale call-train; (**b**) spectrogram of original sperm whale call-train.

According to previous papers [30,31,32,33,34], one can know that sperm whale call pulses are sharp, impulsive, broadband sounds with a well-defined call-train (see Figure 2), and can be produced with a variety of repetition rates, which have been assigned to four main categories: “usual clicks”, “slow clicks”, “creaks”, and “codas”. The most commonly heard sound, “usual clicks”, have an inter-click interval (ICI) of about 0.5 to 1 s; “slow clicks” have an ICI of about 5 to 7 s; “creaks” are series of very rapid clicks with up to 220 clicks per second; and “codas” are short, patterned series of clicks with irregular repetition rates [33]. Multiple call pulses form a call-train. Generally, the source level of sperm whale calls is up to 223 dB. 

Further, the characteristics and laws of the sperm whale call-train can be summarized as follows: (1) Different from those distinct characteristics of conventional CW and LFM sonar signals (the envelope of the CW and LFM pulses are rectangle, the CW is single-frequency signal, and LFM pulse has the linear changed frequency), each call pulse is different from each other in time waveform, frequency domain, and time-frequency distribution; (2) different from the constant time interval between adjacent two pulse of conventional CW and LFM sonar signals, the ICI of sperm whale call pulses changes from 0.0045 (namely 220 clicks per second) to 7 s and in most cases changes from 0.5 s to 2 s [31]; (3) in a short call-train, each category of calls may appear repeatedly, however, in a long call-train, four categories of calls may appear alternately.

If wanting to design a bio-inspired covert (disguised) active sonar strategy, we should preserve and inherit as much as possible the characteristics and laws of sperm the whale call-train and imitate them as much as possible. In the following section, we will firstly describe the construction of the disguised active sonar signal-train.

### 2.2. Construction of the Disguised Active Sonar Signal-Train

Firstly, an original high quality sperm whale call-train of 7 min and 54 s was recorded by a sonobuoy with a 44.1 k sampling rate [35], and a part of the NA signal waveform and spectrogram of the original sperm whale call-train is shown in Figure 2.

**Figure 3:**
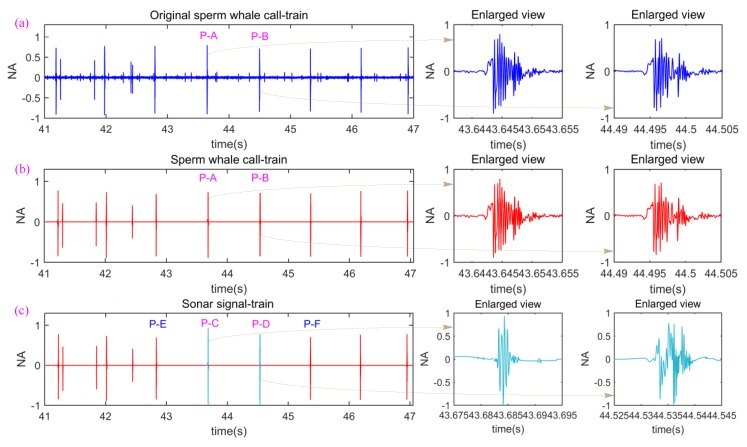
The constructed process of the disguised active sonar signal-train. (**a**) A part of the original sperm whale call-train from the 41st s to 47th s, where the ordinate is the normalized amplitude (NA) of call-train; (**b**) the sperm whale call-train after ocean noise is filtered out and low-energy call pulses are removed; (**c**) the constructed disguised active sonar signal-train.

Then, in order to preserve and inherit as much as possible the characteristics and laws of the sperm whale call-train and imitate them as much as possible, we construct a disguised active sonar signal-train, as shown in Figure 3c. Without loss of generality, firstly, we extract an original sperm whale call-train with a certain time length (please see Figure 3a) from a 7 min and 54 s high quality original sperm whale call-train [35]. It is noteworthy that the original sperm whale call-train received by the sonobuoy includes a series of pulses with different energy and fidelity (Figure 3a), which can be attributed to such factors as original source level, underwater acoustic channel (UAC), direction of sperm whale call pulses, ocean noise, etc. However, it is very complicated and difficult to verify which one, or several, of the factors have the most significant effect on the pulse energy and fidelity. In such case, it may not be the best, but a relatively reliable choice, for us to filter the ocean noise out, remove low-energy call pulses and retain high-energy and high-quality call pulses (namely high signal to noise ratio (SNR)). After we do this, the original sperm whale call-train in Figure 3a is turned into the call-train in Figure 3b. Because only ocean noise and low-energy call pulses are removed, the call-train in Figure 3b almost inherits all characteristics and laws of the original sperm whale call-train in Figure 3a. Next, we use another two original sperm whale call pulses “P-C” and “P-D” to replace the two call pulses “P-A” and “P-B” in Figure 3b, and then obtain Figure 3c. In Figure 3c, the two call pulses “P-C” and “P-D” are used to form a RVMC and are utilized to accomplish the measurement of range and velocity of underwater target, and other call pulses are utilized to serve as maskant so as to disguise the real sonar signal pulses “P-C” and “P-D”. In other words, the constructed disguised active sonar signal-train is composed of real sonar signal pulses and some maskant call pulses.

Comparing Figure 3c with Figure 3a, one can see easily that the constructed disguised active sonar signal-train (Figure 3c) only has two differences with the original sperm whale call-train (Figure 3a). One is that the original sperm whale call-train in Figure 3a contains the ocean noise and low-energy call pulses while the constructed disguised active sonar signal-train in Figure 3c does not. Another is that the two pulses “P-A” and “P-B” are replaced with “P-C” and “P-D”, respectively. Likewise, because only ocean noise and low-energy call pulses are removed and the two sonar pulses “P-C” and “P-D” are also true sperm whale call pulses, the constructed disguised active sonar signal-train (Figure 3c) has very similar characteristics and laws with the original sperm whale call-train in Figure 3a. 

However, in order to construct an efficient, disguised active sonar signal-train and accomplish the accurate measurement of range and velocity of underwater targets with a high concealment, we also need to solve the following four key issues:

(1) How to effectively filter the ocean noise out and remove the low-energy call pulses from the original sperm whale call-train;
(2) What characteristics do sperm whale call pulses have from the perspective of serving as sonar signal pulses? Which call pulses are suitable for sonar signal pulses (such as “P-C” and “P-D”)?
(3) How to measure the range and velocity of underwater targets using the RVMC;
(4) How to further improve the disguised ability of the constructed active sonar signal-train.

Next, in Section 2.3, we will solve the first key issue, the second key issue in Section 2.4, the third key issue in Section 2.5, and the fourth key issue in Section 2.6.

### 2.3. Preprocessing of the Original Sperm Whale Call-Train

As stated in the first paragraph of Section 2.3, in order to obtain high-energy and high-quality call pulses, we need to filter the ocean noise out and remove low-energy call pulses.

Considering that sperm whale call-trains are non-stationary signals [30,31,32,33,34], a wavelet-based denoising method [36,37] is first utilized to clean the noise since the wavelet transform has been proven to be a useful tool for non-stationary signal analysis [36]. Based on the previously described denoising method [37], the denoising process is achieved through the following three steps:

(1) Decompose the mixed signal x(t)=s(t)+n(t) composed of the ocean noise and sperm whale call-train by using wavelet transform; where s(t) and n(t) denote the sperm whale call-train and ocean noise, respectively. More specifically, the noise and sperm whale call-train are decomposed into L levels by discrete wavelet transform using the symlets wavelet-packet.
(2) Use a soft threshold level $t_{n}$ given by an estimator developed by David Donoho [37]
  (1) $$t_{n}=\sigma \sqrt{2\log(n)}$$
  to shrink the wavelet detailed coefficients of the noise. σ is the noise standard deviation and n is the signal length.
(3) The inverse discrete wavelet transform is used to reconstruct the denoised signal.

**Figure 4:**
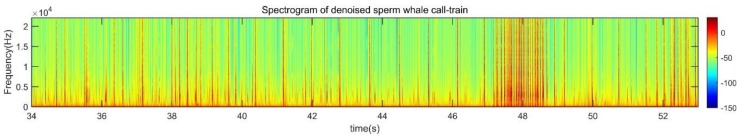
The spectrogram of the denoised sperm whale call-train.

For example, when the orthogonal Symlets wavelet-packet was used with vanishing N=8 (sym8) the wavelet decomposition level is set to 7; the spectrogram of the denoised sperm whale call-train from the 34th to 53rd s is shown in Figure 4. Comparing the Figure 2b and Figure 4, it can be seen that the ocean noise can be filtered out efficaciously.

**Figure 5:**
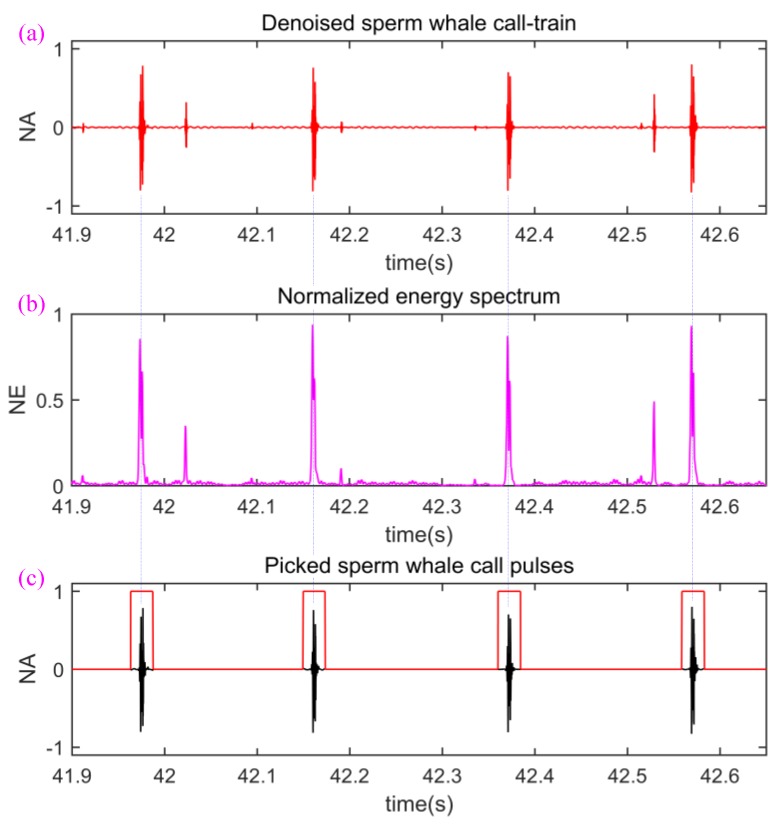
The removal process of low-energy signals. (**a**) Denoised sperm whale call-train; (**b**) NE spectrum; (**c**) picked sperm whale call pulses.

Next, we begin to remove the low-energy call pulse from the denoised sperm whale call-train. Because the sperm whale call pulses have very nice SNR, we intend to utilize the short-term energy spectrum of denoised sperm whale call-train to reach our purpose. The process can be described as:

(1) Firstly, we calculate the short-term energy spectrum [38] of the denoised sperm whale call-train and then normalize the short-term energy spectrum so as to obtain normalized energy (NE) spectrum. Figure 5b gives an intuitive example.
(2) Next, we set a NE threshold value $N_{T}$, then find out all energy peaks which are asked to be more than $N_{T}$, and record the locations in time axis corresponding to call energy peaks. For example, when $N_{T}$ is set to 0.5, the four locations corresponding to four blue dotted lines are recorded in Figure 5.
(3) Then, since the duration of sperm whale call pulses is about 10–20 ms [34], many 22 ms-width rectangle windows are generated and the center of each rectangle windows is aligned to the location of each found energy peak (please see the blue dotted lines between Figure 5b,c).
(4) Finally, all rectangle windows are used to perform the AND operation with the sperm whale call-train in Figure 5a. Because the high and low levels of the rectangle windows are “1” and “0” respectively, the low-energy signals containing low-energy call pulses and residual noise was set to “0” and the high-energy call pulses were not changed. In other words, the low-energy signals containing low-energy call pulses and residual noise are removed and the high-energy call pulses are retained, as shown in Figure 5c.

In next content, we will analyze the characteristics of all high-energy call pulses retained above from the point of serving as sonar signal pulses.

### 2.4. Analysis and Statistics for Sonar Signal Pulses

As is well-known, the range resolution (RR) and velocity resolution (VR) are two very important parameters for evaluating the performance of sonar signals as well as radar signals [39,40]. It is worth noting that the VR has an inverse relationship with Doppler tolerance (DT) [41]; in other words, bad (low) VR means good (high) DT.

When designing the sonar or radar waveforms, the ambiguity function is a very direct and useful tool since it can be utilized to evaluate RR and VR of the designed sonar or radar waveforms. Meanwhile, considering that the sperm whale call pulses are wideband pulses [32,34], the wideband ambiguity function (WAF) [40]

(2) $$\chi (\tau ,\eta )=\sqrt{\eta }\int_{-\infty }^{\infty }s(t)s^{*}(\eta (t-\tau ))dt$$

is used to evaluate the RR and VR of each retained high-energy call pulse; where s(t) denotes each call pulse, τ denotes time delay, η=(c+v)/(c−v)≈1+2v/c, c is the speed of signal propagation, and v is radial relative velocity. 

When η=1, χ(τ,η) can be simplified into

(3) $$\chi (\tau )=\int_{-\infty }^{\infty }s(t)s^{*}(t-\tau )dt$$

Generally, χ(τ) is referred to as the range ambiguity function (RAF) and can be used to examine the RR. Note that χ(τ) is equivalent to the autocorrelation function, and thus high RR is consistent with a sharp autocorrelation peak. 

While τ=0, χ(τ,η) can be simplified into

(4) $$\chi (\eta )=\sqrt{\eta }\int_{-\infty }^{\infty }s(t)s^{*}(\eta (t))dt$$

Generally, χ(η) is called the velocity ambiguity function (VAF) and can be used to examine the VR and the DT [41]. Generally, DT is defined as DT=η−1≈2v/c.

In next section, we will utilize the analysis tools RAF and VAF to evaluate the retained high-energy call pulses and classify them by setting different RR, VR, and DT threshold values. 

Firstly, we use the method mentioned in Section 2.3 to preprocess the 7 min and 54 s high quality original sperm whale call-train recorded by a sonobuoy with a 44.1 ksps sampling rate [35] by using the same parameters with examples shown in Figure 4 and Figure 5. After the preprocessing, 863 high-energy call pulses were obtained. Then, we use χ(τ) and χ(η) to compute the RR, VR, and DT of the 863 call pulses under the condition that c is assumed to be 1500 m/s, and obtained the statistical results shown in Table 1 and Table 2.

Note that in Table 1 and Table 2, the symbols “⊳” and “⊲” mean the “superior (better)” and “inferior (worse)”; for example RR⊳n (or VR⊳n) indicates that the −3 dB width of the RAF (or VAF) of a certain call pulse is less than n. On the contrary, RR⊲n (or VR⊲n) indicates that the −3 dB width of the RAF (or VAF) of a certain call pulse is more than n.

From Table 1, it can be see that all retained call pulses have a very good RR. Even though the RR is expected to be superior to 0.05 m, there still exist 77 call pulses. At the same time, there exists a considerable amount of call pulses whose VR are more than 3 m/s. However, simultaneously considering the RR and VR, the number of satisfactory call pulses declines sharply. For example, when the RR and VR are asked to be superior to 1 m and 2 m/s simultaneously, there only exist two call pulses.

Meanwhile, from Table 2, one can find that the DT of 96.5% of call pulses overmatches 0.0027 (corresponding to 2 m/s VR) and the DT of 20.6% call pulses is superior to 0.0047 (corresponding to 2 m/s VR). Likewise, simultaneously considering the RR and DT, one can find that there still exist 102 call pulses on the conditions that the RR and DT are asked to be superior to 0.5 m and 0.0047 simultaneously.

**Figure 6:**
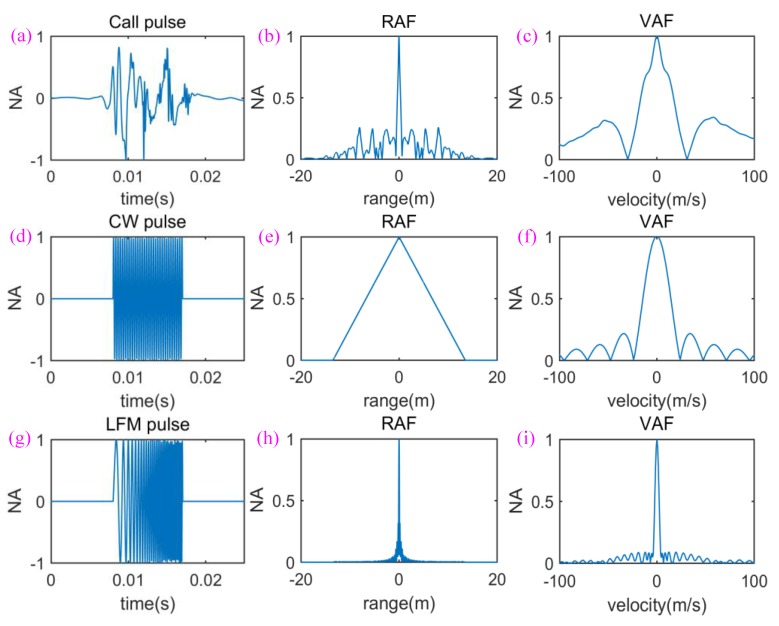
A comparison of RAF and VAF among the sperm whale call pulse, CW pulse, and LFM pulse. (**a**) Waveform of call pulse; (**b**) RAF of the call pulse; (**c**) VAF of the call pulse; (**d**) waveform of CW pulse with 3.5 kHz carrier; (**e**) RAF of the CW pulse; (**f**) VAF of the CW pulse; (**g**) waveform of LFM pulse with a frequency range from 0.5 kHz to 6 kHz; (**h**) RAF of the LFM pulse; (**i**) VAF of the LFM pulse.

As an example, Figure 6a shows a true sperm whale call pulse and its RAF (Figure 6b) and VAF (Figure 6c). For comparison, one CW pulse with the same duration with the true sperm whale call pulse and its RAF and VAF are shown in Figure 6d–f, respectively; and one LFM pulse with the same duration with the true sperm whale call pulse and its RAF and VAF are shown in Figure 6g–i, respectively. On the one hand, comparing Figure 6a,d,g, one can see that the waveform of the sperm whale call pulse has a significant difference compared to the CW and LFM pulses, which is also the reason that it is different from the conventional CW and LFM pulse signals. On the other hand, comparing their RAFs and VAFs, one can find that the sperm whale call pulse has an RAF approximately similar to the LFM pulse and a VAF approximately similar to the CW pulse.

In next Section, we will design effective measurement methods to estimate the range and velocity of underwater targets according to the analysis and statistics results of the sperm whale call pulses mentioned in this Section.

### 2.5. Measurement of Range and Velocity of Underwater Targets

In Section 2.2 we suggested that the sperm whale call pulses, instead of conventional man-made signal waveforms, such as CW or LFM, are used to serve as the sonar signal pulses. Therefore, based on analysis and statistics results mentioned in Section 2.4, we designed a computationally efficient method to measure the range and velocity of underwater targets.

It is well-known that most AUVs are designed with a cruising speed of around 2 m/s as a compromise between long endurance and making reasonable progress, and their maximum movement speeds usually do not exceed 3 m/s [42,43,44]. Meanwhile, as shown previously [45], the submarine’s speed on combat patrols generally does not exceed the so-called “maximum low noise” speed which amounts to nearly 8 knots (4 m/s). According to the range and velocity measurement principle for a traditional sonar system, if we desire to accurately measure the range and velocity of AUVs or submarines and simultaneously distinguish the different ranges and velocities between two adjacent AUVs or two adjacent submarines, the sonar signal pulses must have remarkable RR and VR. However, from Table 1, we have known that although most sperm whale call pulses have outstanding RR, there are few call pulses whose VR is good; for example, there is no call pulse whose VR surpasses 1 m/s. 

Therefore, based on the traditional range and velocity measurement principle, and according to the analysis and statistics about call pulse RR, VR, and DT, it is nearly impossible to accurately measure the velocity of AUVs and distinguish the different velocities between two adjacent AUVs by utilizing traditional velocity measurement principles and single sperm whale call pulses. At the same time, when the relative speed between two adjacent submarines is not more than 2 m/s, it also is nearly impossible to accurately distinguish the different velocities between two adjacent submarines.

In addition, there exists a great deal of call pulses whose RR and DT are superior to 0.5 m and 0.0047 simultaneously, and DT = 0.0047 is corresponding to 3.5 m/s, which is more than the maximum movement speed of conventional AUVs. Therefore, according to the analysis and statistics about call pulse RR, VR, and DT, we designed a two-step measurement (TSM) method to estimate the range and velocity of targets.

**Figure 7:**
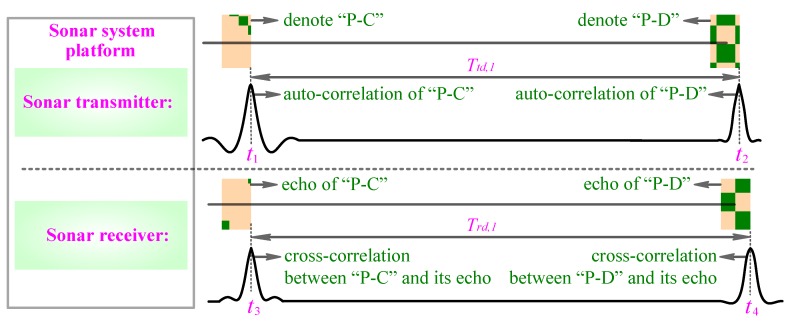
The measurement principle of the range and velocity of underwater targets.

**Figure 8:**
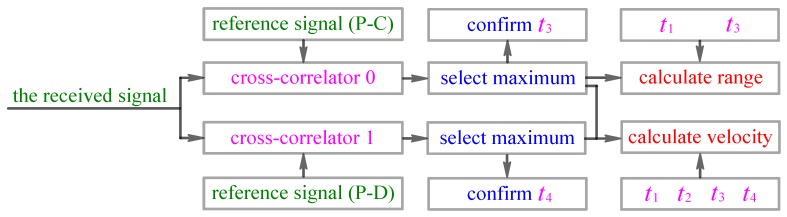
The principle of the sonar receiver.

Firstly, to measure the range of targets, we use a single call pulse, which is selected from Table 2 and simultaneously has remarkable RR (e.g., superior to 0.5 m) and good DT (e.g., superior to 0.0047), to serve as the range measurement sonar waveform. Then we use this call pulse to replace a certain call pulse in the preprocessed sperm whale call-train and achieve the range measurement of targets by using the conventional sonar range measurement principle. For example, we utilize the selected call pulse P-C (see Figure 3c), which has remarkable RR and good DT, to replace the call pulse P-A (see Figure 3b) of the preprocessed call-train so as to let it serve as a sonar waveform to accomplish the range measurement of targets. Further, assume that the time at which the P-C is sent out is $t_{1}$, the time at which the P-D is sent out is $t_{2}$, the time at which the echo of P-C is received is $t_{3}$, and the time at which the echo of P-D is received is $t_{4}$ (please see Figure 7). Because the sonar transmitter and receiver are integrated in the same sonar system platform, the time $t_{1}$ is known as the sonar receiver. Then, the sonar receiver can confirm $t_{3}$ by selecting a maximum cross-correlation peak of the cross-correlator 0 (please see Figure 8). Based on $t_{1}$ and $t_{3}$, the range R of the underwater target can be calculated through $R=c\cdot (t_{3}-t_{1})/2$.

Secondly, in order to measure the velocity of targets, we use another call pulse (such as pulse P-D) again, which is selected from Table 2 and simultaneously has remarkable RR (e.g., superior to 0.5 m) and good DT (e.g., superior to 0.0047), to replace another call pulse (such as pulse P-B) in the preprocessed sperm whale call-train. Then, we set a known time difference $T_{td}$ between two selected call pulses and use the two call pulses to form a RVMC.

At the same time, these two selected call pulses must also satisfy another condition, that is to say, that there is as small a cross-correlation peak value as possible between them to avoid confusing them at the receiver. In fact, this condition is easy to be satisfied via cross-correlation calculation and further screening. For example, when the normalized cross-correlation peak is asked to be 0.3, 26 RVMCs can be selected from Group 4 in Table 2.

Next, without loss of generality, we assume that the underwater platform with active sonar is static (zero velocity), targets are moving at a relative speed v (for most AUVs, v is not more than 3 m/s), and the time difference between call pulses P-A and P-B in Figure 3a is $T_{td,1}$.

Then we use the two selected call pulses P-C and P-D to replace the two adjacent call pulses P-A and P-B with the same time difference $T_{td,1}$ so as to form a RVMC. When P-C and P-D pulses are reflected back by targets with a velocity v, the cross-correlation peaks between the transmitted and the received sonar waveforms will hardly be affected by the moving speed v of the targets because the DTs of both the P-C and P-D pulses precede 0.0047 (corresponding to 3.5 m/s, it is more than the relative speed v of most AUVs). But, in this case, the time difference $T_{td,1}$ between the P-C and P-D pulses will be compressed or stretched to $T_{rd,1}$ owing to the Doppler Effect between the underwater platform with active sonar and the moving targets; this is to say, $T_{rd,1}$ is the time difference between the echo of P-C and the echo of P-D. Therefore, we can utilize the change of the time difference between $T_{td,1}$ and $T_{rd,1}$ to compute the stretch or compression factor η of the sonar waveform through,

(5) $$\eta =T_{td,1}/T_{rd,1}$$

and then based on η=(c+v)/(c−v)≈1+2v/c, to calculate the relative speed v of moving targets through $v\approx (T_{td,1}/T_{rd,1}-1)\cdot c/2$. Further, from Figure 8, one can know $T_{td,1}=t_{2}-t_{1}$, $T_{rd,1}=t_{4}-t_{3}$. And, the sonar receiver can confirm $t_{4}$ by selecting maximum cross-correlation peak of the cross-correlator 1 (please see Figure 8). Finally, the relative speed v of moving targets can be estimated through $v\approx [(t_{2}-t_{1})/(t_{4}-t_{3})-1]\cdot c/2$.

It is noteworthy that because those call pulses have high DT (corresponding to $v_{1}$), their auto-correlation peaks are hardly affected by the relative speed $v_{2}$ ($v_{2}<v_{1}$) between the underwater platform with active sonar and targets. That is why we screen those call pulses with good auto-correlation and high DT.

It is worth mentioning that the correlator is a most reliable way to measure the time difference $T_{rd,1}$, and it is a very computationally efficient tool. Thus it is very beneficial to practical applications.

It should be noted that in order to prevent the echoed signals of sonar pulses P-C and P-D from be influenced by the ones of call pulses P-E and P-F, some protection time should be arranged between the call pulses P-E and P-C, P-D and P-F, according to the requirements of practical applications. As previously mentioned, we use the sonar pulses (e.g., pulses P-C and P-D) to replace the original sperm whale call pulses (e.g., P-A and P-B). At the same time, because the most ICIs between sperm whale call pulses vary in [0.5, 7] s [33], the time difference (namely ICI) $T_{td}$ between two sonar pulses is not constant but is changeable as the time difference (namely ICI) between two replaced call pulses changes. 

In addition, the echoes of sonar signals are received by the hydrophone (acoustic sensor) or hydrophone array (receiver) installed in sonar system platforms (please see Figure 7). Because the receiving process of sonar signals is a passive and covert one in itself, in this paper, we do not discuss the bio-inspired design of the sonar receiver.

### 2.6. Improving of the Disguised and Covert Ability

Firstly, if the same RVMC (sonar signal pulses) is sent all the time, such as P-C and P-D in Figure 3c, a distinguishable call pulses repetitive feature in the whale call-train with RVMC may be generated, which is not beneficial to its camouflage ability. Therefore, the selected multiple RVMCs (such as 26 RVMCs in Section 2.5) may be used to serve as sonar signal pulses and then are sent according to a known and random sending order.

Secondly, likewise, the time difference $T_{td,1}$ between two selected call pulses should also be changed in the time range [0.0045, 7] s, especially [0.5, 2] s [18] so that a distinguishable time difference repetitive feature does not be generated.

Finally, although this paper does not utilize the low probability-of-detection (LPD) design, which is used by conventional covert sonar design methods, but instead uses the camouflage to accomplish covert detection, when detecting targets in a small distance range, the underwater platform with active sonar can transmit the sonar signal pulses with low SNR, which is very beneficial to improve concealment ability from a LPD perspective.

## 3. Discussions

What is noteworthy is that because the original sperm whale call pulses (such as P-C and P-D in Figure 3) are used for sonar signal pulse and original sperm whale call-train is utilized for maskant, so as to camouflage the real sonar signal pulses, the sperm whale call-train with sonar signal pulses almost entirely inherits the wave shape, frequency distribution, time-frequency distribution, and ICI characteristics and laws of the original sperm whale call-train, and thus has very good concealment ability.

However, because there might only be a small number of RVMCs in a certain time length sperm whale call-train with sonar signal pulses and all other call pulses are maskant (camouflage call pulses), lots of signal emission energy will be consumed by the maskant (camouflage call pulses), which needs to be faced in practical application.

In addition, when the speed of underwater targets is larger and more than the cruising speed of submarines (4 m/s), as mentioned in Section 2.5, the measurement accuracy, range, and velocity of underwater targets will decline due to the limited DT of sperm whale call pulses. In future work, we will explore to solve this issue.

## 4. Simulations and Experiments

### 4.1. Disguised Ability of Constructed Sonar Signal-Train

**Figure 9:**
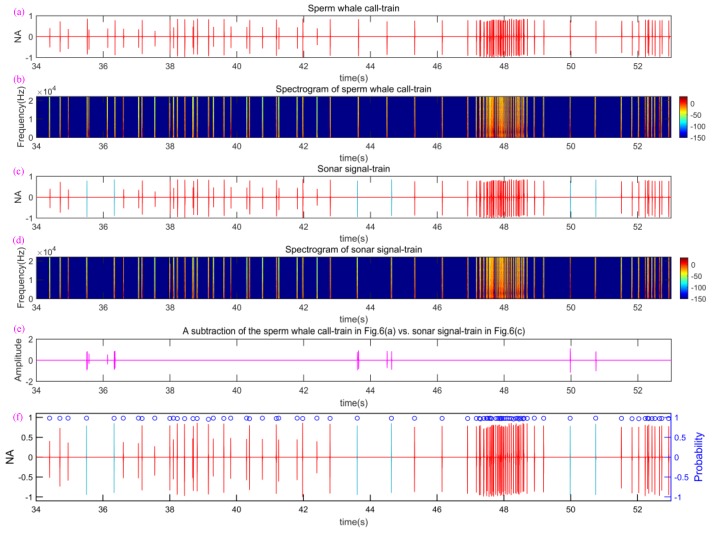
Comparison of waveform and spectrogram of both sperm whale call-train and sonar signal-train, and the results classified by NNBC. (**a**) A part of denoised sperm whale call-train from 34th to 53rd s; (**b**) the spectrogram of sperm whale call-train corresponding to (**a**,**c**) sonar signal-train embed by RVMC; (**d**) the spectrogram of sperm whale call-train corresponding to (**c**,**e**) a subtraction of the sperm whale call-train in Figure 9a vs. sonar signal-train in (**c**,**f**) the classification and recognition probability of sperm whale call pulses; the blue “o” denotes the probability that pulses are classified into sperm whale call ones.

In this experiment, without loss of generality, we selected a preprocessed sperm whale call-train (such as from the 34th to 53rd s, see Figure 9a), and then used three different RVMCs selected in Section 2.5 to replace six original call pulses so as to construct a sonar signal-train, as is shown in Figure 9c. 

Then the spectrograms of the preprocessed sperm whale call-train and constructed sonar signal-train are shown in Figure 9b,d respectively. Comparing Figure 9a,c and Figure 9b,d, one can hardly find the difference and distinction between Figure 9a,c and Figure 9b,d. This demonstrates the constructed sonar signal-train has a very high disguised ability in term of wave shape, frequency distribution, time-frequency distribution, and ICI characteristics and laws. 

Further, we utilized the current advanced underwater classification and recognition method, the Neural network-based classification (NNBC) method described previously [46], widely used by underwater reconnaissance systems, to evaluate the disguised and covert ability of a constructed sonar signal-train. We utilized the five order polynomials to fit the ridge of the TFS of call pulses, and then five coefficients and residual error of the five order polynomials were used as feature parameters to train the neural network. Afterwards, 200 sperm whale clicks were used to train the neural network. The neural network was then used to recognize and classify the preprocessed sperm whale call-train of 7 min and 54 s composed of 863 call pulses (containing sonar signal pulses). The subtraction of the sperm whale call-train in Figure 9a vs. sonar signal-train in Figure 9c was used to evaluate the similar degree between the sperm whale call-train and sonar signal-train, as shown in Figure 9e. It can be seen that except for three section signals embedded by three RVMCs, other signal-trains of the sonar signal-train were the same as the sperm whale call-trains, which demonstrates that there exits only a very small difference and thus good camouflage ability can be achieved. The results show that the classification and recognition probability of sperm whale call pulses are 100%, and an intuitive example of the results is shown in Figure 9f. The right ordinate scale in Figure 9f indicates the normalized output value of the neural network, which can be generally considered as the recognition probability. The classification and recognition results show that the neural network always decides sonar signal pulses into the sperm whale call pulses (namely ocean noise [27,47]). Therefore, the constructed disguised sonar signal-train achieves very excellent camouflage capacity.

### 4.2. Ouput Power Comparison

**Figure 10:**
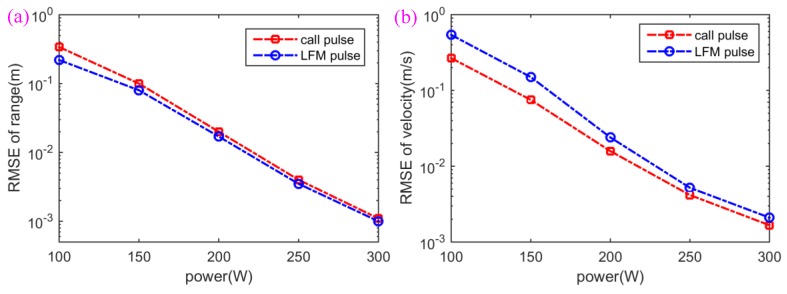
Output power comparison when the sperm whale call pulse and LFM pulse are used to serve as the sonar signal waveforms for range measurement of the underwater target. (**a**) RMSE of range, (**b**) RMSE of velocity.

In this simulation, the range and velocity estimation performance of the proposed method is compared with the traditional method [48] as change of the output power of the transmitted sonar signals. Please note that considering that the output power of the sonar transmitter is an input condition rather than an output condition results in the range and velocity estimation, it is unnecessary if only the output power is given. Therefore, from another perspective, we compare the output power between the proposed method and the traditional method; that is to say, we compare the range and velocity performance of the proposed method and the traditional method under the condition that the output power is the same to each other. Because it has outstanding RR and VR, an LFM pulse [48] is used widely in traditional sonar and radar detection methods. Thus, in this simulation, a single LFM pulse was used for the traditional method, and is utilized to serve as a sonar waveform to estimate the range and velocity of the target, and its estimation performance is compared with the proposed method in this paper. In this simulation, the sperm whale call pulse in Figure 6a is used for the proposed method and the LFM pulse in Figure 6g is used for the traditional method. The distance (namely range) between the transmitter and the target is set to 1 km, the depths of the transmitter and the target are 100 m, the reflection coefficient of the target is one, and the underwater acoustic channel (WATTCH) model as described previously [49] is used to simulate the practical oceanic environment. A set of eigen-rays is then generated through BELLHOP [50,51], and they represent all of the significant contributing acoustic paths between the source and the receiver [52]. The target moves at a relative speed of v=2 m/s away from the sonar transmitter. The root-mean-square errors (RMSEs) [53] of range and velocity estimation results (ERs) with 100 independent Monte Carlo runs [53] are shown in Figure 10 as the change of output power of the sonar transmitter. It can be seen from Figure 10a that the range estimation performance of the traditional method using the LFM pulse slightly overmatches that of the proposed method using the sperm whale call pulse, which is because the LFM pulse has better RR than the sperm whale call pulse. On the other hand, it can be seen from Figure 10b that the velocity estimation performance of the proposed method obviously exceeds that of the traditional method. This is because that the traditional method utilizes the Doppler sensitivity of a single LFM pulse to measure velocity of the target, while the proposed method utilizes both the Doppler invariance and the excellent time resolution (corresponding to excellent RR) to measure the velocity of the target. It is worth mentioning that the sperm whale call pulse used as sonar waveform can obtain covertness through camouflage, while the LFM cannot.

### 4.3. Efficiency of Underwater Targets Detection

**Figure 11:**
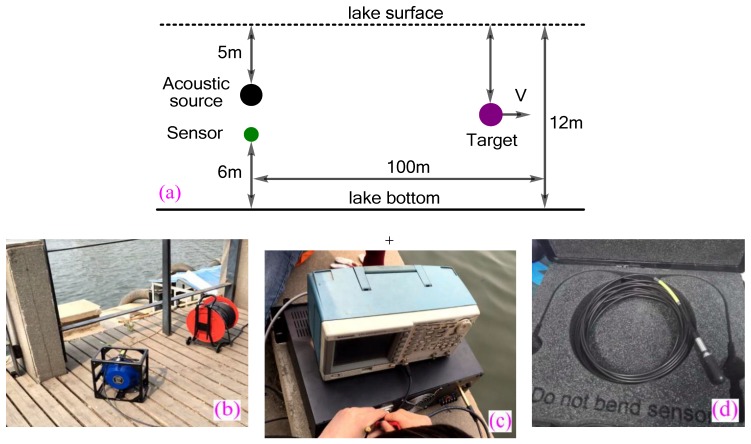

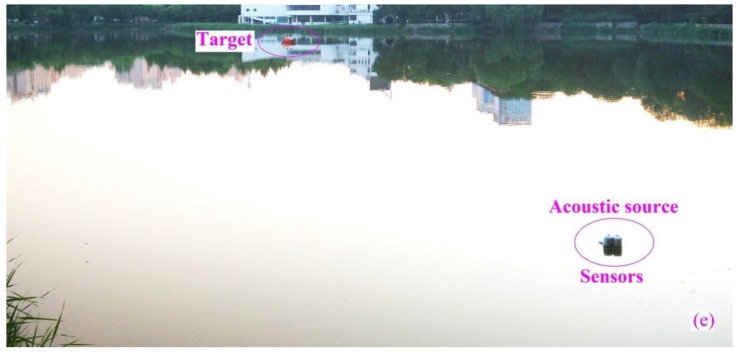
The experimental scenario and relevant parameters. (**a**) The experimental model; (**b**) the broadband acoustic source; (**c**) the corresponding power amplifier; (**d**) the broadband hydrophone; (**e**) the experiment field.

In order to verify the efficiency of the presented active sonar strategy for range and velocity estimation of underwater targets, a lake experiment was set up, as shown in Figure 11a–e. A LL916 projector (Lubell Labs Inc., Columbus, OH, USA) (operating bandwidth 200 Hz–23 kHz) was used as an acoustic source (Figure 11b) and the corresponding power amplifier is given in Figure 11c. A TC4013 hydrophone (Teledyne Reson Inc., Thousand Oaks, CA, USA) (frequency range 1 H–170 kHz) was used as echo receiving sensor (Figure 11d). The experiment was conducted in QinLian Lake in China with 12 m in depth.

**Figure 12:**
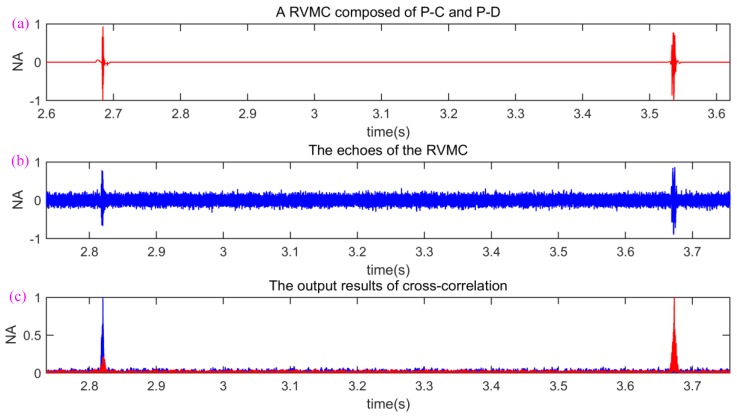
An example of lake experimental results. (**a**) A transmitted RVMC composed of P-C and P-D; (**b**) the echoes of the transmitted RVMC; (**c**) the output results of cross-correlation.

**Figure 13:**
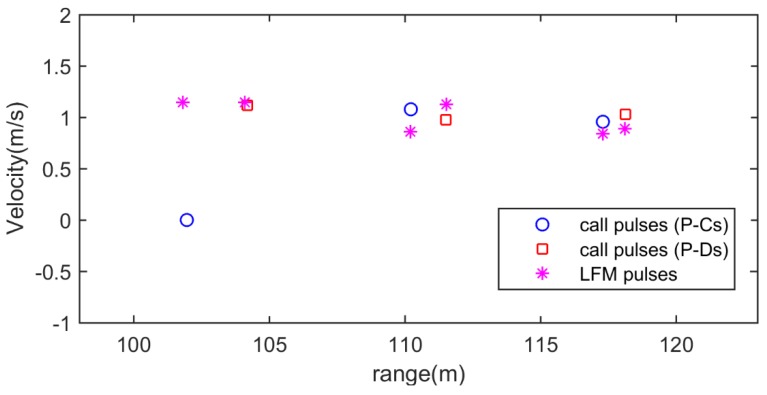
The estimated locus of ERs of range and velocity as time changes.

The measured sound-speed profile was almost constant. A barrel with 1 m length, 0.8 m width, and 0.6 m height was used as the target. The barrel was dragged by a boat and its speed was about 1 m/s. Other experimental parameters were shown in Figure 11. The sonar signal-train in Figure 3c was sent three times repeatedly to estimate the range and velocity of target. Figure 12 shows an example of lake experimental results. The transmitted sperm whale call pulses P-C and P-D (they form a RVMC, see Figure 12a) were reflected by the target, and then their echoes arrived in the received sensor, as shown in Figure 12b. The matched filter outputs (here cross-correlation is used) between pulse P-C and its echo, and between pulse P-D and its echo are shown in Figure 12c. The ER of range and velocity of the target are shown in Figure 13. Because both sonar signal pulses P-C and P-D in Figure 3c can be used to measure the range of target, and the RVMC composed of them can be utilized to measure the velocity, six ERs were obtained. The first ER (ER-1) only contained range instead of velocity (0 m/s), which was because only one pulse cannot estimate the velocity of target based on the velocity measurement principle described in Section 2.5. After the echo of the second sonar signal pulse P-D was received, ER-2 of range and velocity were obtained simultaneously. Please note that the range estimate denoted by ER-1 is different from the one contained by ER-2, which is because the target is moving in the time difference between pulses P-C and P-D. Similarly, because the sonar signal pulses P-Cs sent in the next two times also cannot estimate the velocity of target, the two ERs, namely ER-3 and ER-5 inherit the velocity estimates of ER-2 and ER-4, respectively. Likewise, owing to incessant movement of target, the range ER corresponding to each sonar signal pulse increases gradually. Most important of all, satisfactory range and velocity ERs were obtained in this experiment, which demonstrates the efficiency of the presented bio-inspired covert (disguised) active sonar strategy. 

For comparison, another state of the art method [48] using the LFM pulse as sonar pulse was also executed in the lake experiment. In order to ensure the same experimental conditions, we used two same LFM pulses in Figure 6g to replace P-C and P-D pulses in Figure 3c to form a new sonar signal-train, and then sent this new sonar signal-train three times repeatedly to estimate the range and velocity of target. Please note that due to good RR and VR of the LFM pulse, the range and velocity of the target were estimated simultaneously through single LFM pulse. Based on a state of the art method [48], the ERs of range and velocity of the target are also shown in Figure 13. It can be seen from Figure 13 that the range ERs of the proposed method are very close to that of the state of the art method [48]; however, the variance of velocity ERs of the state of the art method [48] are larger than the proposed method, which is consistent with the simulation results in Figure 6.

## 5. Conclusions

In this paper, a novel bio-inspired covert (disguised) active sonar strategy is presented. Not using the conventional man-made sonar waveforms, such as CW, LFM, etc., and not using the bionic man-made sonar waveforms, such as the double down-chirp waveform described previously [19], the true sperm whale call pulses are used to serve as sonar waveforms so as to ensure the camouflage ability of sonar waveforms. The RVMC is constructed and the corresponding range and velocity estimation methods were developed to measure the range and velocity of targets. To improve the camouflage ability of the sonar signal-train, the RVMC are inserted into the true sperm whale call-train. Finally, experiment results demonstrate the disguised and covert ability and estimation performance of the disguised sonar signal-train. In future research, some similar, in-depth and interesting work can be done. For example, in next work, similar to a previously completed work [26], we will use the whale call pulses and combine some human made manipulations, such as man-made ICI, to reach some different goals, such as increasing the transmission rate, and achieving a more accurate localization, and so on.

## Figures and Tables

**Table 1:** The number of call pulses under different RR and VR (or DT) threshold values.

**RR (m) or** τ **(ms)**		**Number**	**VR (m/s) or DT**	**Number**
RR ⊳ 1.50 or τ ⊳ 1.000		863	VR ⊳ 1.0 or DT ⊲ 0.0013	0
RR ⊳ 1.00 or τ ⊳ 0.667		839	VR ⊳ 2.0 or DT ⊲ 0.0027	16
RR ⊳ 0.50 or τ ⊳ 0.333		620	VR ⊳ 3.0 or DT ⊲ 0.0040	198
RR ⊳ 0.10 or τ ⊳ 0.067		495	VR ⊳ 4.0 or DT ⊲ 0.0053	812
RR ⊳ 0.05 or τ ⊳ 0.033		77	VR ⊳ 5.0 or DT ⊲ 0.0067	863
**Group**	**Threshold value**			**Number**
1	{RR ⊳ 1.0 m, VR ⊳ 3.0 m/s} or {τ ⊳ 0.667 ms, DT ⊲ 0.0040}			189
2	{RR ⊳ 1.0 m, VR ⊳ 2.5 m/s} or {τ ⊳ 0.667 ms, DT ⊲ 0.0033}			46
3	{RR ⊳ 1.0 m, VR ⊳ 2.0 m/s} or {τ ⊳ 0.667 ms, DT ⊲ 0.0027}			2
4	{RR ⊳ 0.5 m, VR ⊳ 2.0 m/s} or {τ ⊳ 0.333 ms, DT ⊲ 0.0027}			0
5	{RR ⊳ 1.5 m, VR ⊳1.0 m/s} or {τ ⊳ 1.000 ms, DT ⊲ 0.0013}			0

**Table 2:** The number of call pulses under different RR and DT (or VR) threshold values.

RR (m) or τ (ms)		Number	VR (m/s) or DT	Number
RR ⊳ 1.50 or τ ⊳ 1.000		863	VR ⊲ 2.0 or DT ⊳ 0.0027	833
RR ⊳ 1.00 or τ ⊳ 0.667		839	VR ⊲ 2.5 or DT ⊳ 0.0033	607
RR ⊳ 0.50 or τ ⊳ 0.333		620	VR ⊲ 3.0 or DT ⊳ 0.0040	308
RR ⊳ 0.10 or τ ⊳ 0.067		495	VR ⊲ 3.5 or DT ⊳ 0.0047	178
RR ⊳ 0.05 or τ ⊳ 0.033		77	VR ⊲ 4.0 or DT ⊳ 0.0053	65
Group	Threshold value			Number
1	{RR ⊳ 1.5 m, VR ⊲ 4.0 m/s } or {τ ⊳ 1.000 ms, DT ⊳ 0.0053}			65
2	{RR ⊳ 1.5 m, VR ⊲ 3.5 m/s} or {τ ⊳ 1.000 ms, DT ⊳ 0.0047}			178
3	{RR ⊳ 1.0 m, VR ⊲ 3.5 m/s} or {τ ⊳ 0.667 ms, DT ⊳ 0.0047}			170
4	{RR ⊳ 0.5 m, VR ⊲ 3.5 m/s} or {τ ⊳ 0.333 ms, DT ⊳ 0.0047}			102
5	{RR ⊳ 0.1 m, VR ⊲ 3.5 m/s} or {τ ⊳ 0.067 ms, DT ⊳ 0.0047}			86
6	{RR ⊳ 0.1 m, VR ⊲ 3.0 m/s} or {τ ⊳ 0.067 ms, DT ⊳ 0.0040}			52
